# Supplementary material for: A Case of Curative Treatment with Apatinib and Camrelizumab Following Liver Resection for Advanced Hepatocellular Carcinoma
Source: Int J Mol Sci. 2023 Aug 30;24(17):13486. doi: 10.3390/ijms241713486 (PMC10487462; doi:10.3390/ijms241713486)
Supplement: Supplementary file 1 [file ijms-24-13486-s001.zip › ijms-2487688-supplementary.pdf]

A case of curative treatment of apatinib associated calibizumab following liver resection for advanced hepatocellular carcinoma

Methods

Genomic characteristics of the case patient before apatinib and calibizumab treatments

In order to clarify the genomic characteristics of the HCC case patient, we made a visual analysis of the CNV and SNP of the case patient using the trackViewer package[1]. Then, in order to explore the Kyoto Encyclopedia of genes and genes (KEGG) pathway which involved in CNV and SNP, we used iSubpathway [2] in R package to the sub pathway enrichment. Post-based on sub-pathway enrichment analysis, the potential mechanisms where CNV and SNP may be involved in promoting the occurrence and development of liver cancer are explored.

58 TCGA LIHC patients with genome similar to the case patient were selected as control

We first download data from TCGA database using TCGAbiolinks [3] in R package, and use GISTIC2 [4] software to identify the CNV region, so as to get the CNV variation region of typical TCGA- LIHC and HCC case patient for comparison. At the same time, we also got the mutation of TCGA LIHC patients and HCC case patient, and used maftools [5] in R

package to show the SNP of the LIHC patients in TCGA and HCC case patient. We used TCGA-LIHC database to carry out chi-square test with HCC case patient respectively to get p value. When $p < 0.05$, the difference between case patients and TCGA-LIHC samples was significant. When p value > 0.05 meaning no significant differences of SNP between them.

Transcriptome characteristics of the case patient after apatinib and calibizumab treatments compared with 58 TCGA-HCC patients as control.

Compared with LIHC patients with similar genomes in TCGA clinical cohort, we used R package DESeq2 [6] to analyze the differential expression of the standardized gene expression profile of the HCC case patient to obtain the differentially expressed gene in HCC case patient. Then, the KEGG pathway enrichment analysis was performed using clusterProfiler [7], so as to obtain KEGG pathway with significant participation of differentially expressed mRNA. Based on previous work [8] combined with CIBERSORT[9] and MCP Counter [10], the immune-related gene set was obtained. Then, the Gene set variation analysis (GSVA) [11] was applied using immune-related gene set, obtained the GSVA score of immune-related gene set. The difference of immune infiltration between HCC case patient and TCGA LIHC clinical cohort

with genomic similarity was compared by the GSVA score of the immune related gene set.

Reference

1. Ou, J. and L.J. Zhu, *trackViewer: a Bioconductor package for interactive and integrative visualization of multi-omics data.* Nat Methods, 2019. **16**(6): p. 453-454.
2. Li, C., et al., *Subpathway-GM: identification of metabolic subpathways via joint power of interesting genes and metabolites and their topologies within pathways.* Nucleic Acids Res, 2013. **41**(9): p. e101.
3. Colaprico, A., et al., *TCGAbiolinks: an R/Bioconductor package for integrative analysis of TCGA data.* Nucleic Acids Res, 2016. **44**(8): p. e71.
4. Mermel, C.H., et al., *GISTIC2.0 facilitates sensitive and confident localization of the targets of focal somatic copy-number alteration in human cancers.* Genome Biol, 2011. **12**(4): p. R41.
5. Mayakonda, A., et al., *Maftools: efficient and comprehensive analysis of somatic variants in cancer.* Genome Res, 2018. **28**(11): p. 1747-1756.
6. Love, M.I., W. Huber, and S. Anders, *Moderated estimation of fold change and dispersion for RNA-seq data with DESeq2.* Genome Biol, 2014. **15**(12): p. 550.
7. Yu, G., et al., *clusterProfiler: an R package for comparing biological themes among gene clusters.* OMICS, 2012. **16**(5): p. 284-7.
8. Subramanian, A., et al., *Gene set enrichment analysis: a knowledge-based approach for interpreting genome-wide expression profiles.* Proc Natl Acad Sci U S A, 2005. **102**(43): p. 15545-50.
9. Xiao, Y., et al., *Multi-Omics Profiling Reveals Distinct Microenvironment Characterization and Suggests Immune Escape Mechanisms of Triple-Negative Breast Cancer.* Clin Cancer Res, 2019. **25**(16): p. 5002-5014.
10. Newman, A.M., et al., *Robust enumeration of cell subsets from tissue expression profiles.* Nat Methods, 2015. **12**(5): p. 453-7.
11. Herranz Amo, F., et al., *[Cysts of the seminal vesicles: apropos of a case].* Actas Urol Esp, 1987. **11**(2): p. 210-3.
